# The numerical stability of transformation-based CT ventilation

**DOI:** 10.1007/s11548-016-1509-x

**Published:** 2017-01-05

**Authors:** Edward Castillo, Richard Castillo, Yevgeniy Vinogradskiy, Thomas Guerrero

**Affiliations:** 1 Department of Radiation Oncology, Beaumont Health Systems, Royal Oak, MI USA; 2 Department of Computational and Applied Mathematics, Rice University, Houston, TX USA; 3 Department of Radiation Oncology, University of Texas Medical Branch, Galveston, TX USA; 4 Department of Radiation Oncology, University of Colorado, Denver, CO USA

**Keywords:** Computed tomography, Ventilation, Deformable image registration, Functional image analysis

## Abstract

**Abstract:**

Computed tomography (CT)-derived ventilation imaging utilizes deformable image registration (DIR) to recover respiratory-induced tissue volume changes from inhale/exhale 4DCT phases. While current strategies for validating CT ventilation rely on analyzing its correlation with existing functional imaging modalities, the numerical stability of the CT ventilation calculation has not been characterized.

**Purpose:**

The purpose of this study is to examine how small changes in the DIR displacement field can affect the calculation of transformation-based CT ventilation.

**Methods:**

First, we derive a mathematical theorem, which states that the change in ventilation metric induced by a perturbation to single displacement vector is bounded by the perturbation magnitude. Second, we introduce a novel Jacobian constrained optimization method for computing user-defined CT ventilation images.

**Results:**

Using the Jacobian constrained method, we demonstrate that for the same inhale/exhale CT pair, it is possible to compute two DIR transformations that have similar spatial accuracies, but generate ventilation images with significantly different physical characteristics. In particular, we compute a CT ventilation image that perfectly correlates with a single-photon emission CT perfusion scan.

**Conclusion:**

The analysis and experiments indicate that while transformation-based CT ventilation is a promising modality, small changes in the DIR displacement field can result in large relative changes in the ventilation image. As such, approaches for improving the reproducibility of CT ventilation are still needed.

## Introduction

Deformable image registration (DIR) methods compute a spatial transformation that describes the apparent motion depicted by a pair of images [1]. Medical imaging applications, such as radiation dose accumulation [2, 3] and intensity variation analysis [4], rely on DIR algorithms to link corresponding voxel locations. Other applications, such as brain morphometric analysis [5, 6] and cardiac strain rate imaging [7, 8], utilize DIR-measured structural changes to quantify the effects of disease and injury.

Computed tomography (CT)-derived ventilation imaging is based on employing DIR to infer local tissue volume changes, induced by respiratory motion, from inhale/exhale lung CT image pairs [9, 10]. Moreover, CT ventilation is an ideal analysis tool for investigating the effects of radiotherapy on pulmonary function (see [11, 12] for example), since CT ventilation can be computed directly from routine simulation (treatment planning) 4DCT.

There are two strategies for computing CT ventilation. *Intensity-based* ventilation employs the formulation introduced in [13] to estimate local volume changes from the Hounsfield units (HU) of DIR-linked voxel locations [9, 10, 14]. *Transformation-based* ventilation, which is the focus of this study, is derived from multivariate calculus and utilizes the *Jacobian* of the DIR spatial transformation [10, 15]. The *Jacobian factor* of a spatial transformation is defined as the determinant of the transformation’s first derivative (Jacobian) and represents the magnification factor for volumes under the transformation [16]. For simplicity, the Jacobian factor is often referred to as simply the Jacobian.

The goal in developing and understanding CT ventilation is ultimately to employ it in the clinical setting. As such, validation is an important and active area of research. Previous validation strategies have focused almost exclusively on demonstrating a correlation with an established functional imaging modality. For example, comparison studies based on SPECT ventilation [10], SPECT perfusion [17], positron emission tomography (PET) imaging [18], and $\hbox {He}^{3}$ hyperpolarized MRI [19] have all successfully demonstrated varying degrees of spatial correlation with CT ventilation. A related problem, considering the modality’s reliance on DIR, is assessing the spatial accuracy of the DIR algorithm used to compute CT ventilation [20]. Not surprisingly, previous studies have demonstrated that CT ventilation is in fact sensitive to DIR algorithm [21, 22] and to issues affecting DIR algorithm performance, such as 4DCT phase-binning artifacts [23].

DIR validation is itself an active area of research [24]. Spatial accuracy assessment of DIR solutions based on large sets of expert-determined landmark point pairs has been shown to be a statistically robust and straightforward framework [20, 25]. The approach has been utilized within many studies to validate novel DIR algorithm performance (see [26–30] for example). Though there is no universal standard that defines an acceptable DIR spatial accuracy for all situations, in the context of inhale/exhale CT image pairs, DIR algorithms are expected to produce spatial accuracies on the order of the voxel dimensions [31]. Therefore, the pertinent issue for CT ventilation is the degree to which the ventilation image can vary with respect to different DIR solutions within this accuracy range. Or put another way, we seek an answer to the following question: will two DIR solutions for the same CT inhale/exhale image pair generate similar CT ventilation images if both DIR solutions have subvoxel spatial accuracy?

The purpose of this study is to (1) examine the numerical instability inherent to transformation-based CT ventilation and (2) demonstrate how two (or several) DIR transformations with similar spatial accuracies can generate corresponding ventilation images with significantly different physical characteristics. To do this, we first mathematically analyze how perturbing a single displacement vector affects the ventilation calculation. The analysis indicates that, for a single voxel, the maximum possible magnitude change in ventilation metric that can be induced by perturbing a single displacement vector is on the order of the perturbation magnitude. Thus, changing a single displacement by one voxel can result in a magnitude Jacobian change of 1.0. In order to demonstrate the repercussions of this result, we introduce a novel post-DIR processing method for computing a spatial transformation with “user-defined” Jacobian values. Given an initial DIR spatial transformation, this numerical tool allows us to compute a similar transformation that has Jacobian values equal to a pre-specified target Jacobian image. Using this tool, *we demonstrate that for the same inhale/exhale CT pair, it is possible to compute two (or several) ventilation images that have significantly different physical characteristics, despite being generated from DIR solutions with similar spatial accuracies.* DIR spatial accuracy is measured using expert-determined landmark point pairs and imaging data made publically available on the www.dir-lab.com website [20]. The rest of the paper is organized as follows: “Transformation-based CT ventilation” section describes transformation-based CT ventilation and how it is computed numerically. In “Perturbation analysis of the ventilation metric” section, the mathematical bound describing how perturbing a single displacement vector effects the ventilation metric is derived. “Computing spatial transformations with user-defined Jacobian values” section introduces our novel post-DIR processing method for computing spatial transformations with user-defined Jacobian values. Finally, the numerical experiments are presented in “Numerical experiments” section and discussed in “Discussion” section.

## Transformation-based CT ventilation

DIR determines a spatial transformation, $\phi (\mathbf{x}):{\mathbb {R}}^{3}\rightarrow {\mathbb {R}}^{3}$, that maps the image content from a reference image onto a target image. The transformation is often defined in terms of a displacement field:

1 $$\begin{aligned} \phi (\mathbf{x})=\mathbf{x}+\mathbf{d}(\mathbf{x}), \end{aligned}$$

where $\mathbf{d}(\mathbf{x})=\left[ {d^{(1)}(\mathbf{x}),\;\;d^{(2)}(\mathbf{x}),\;\;d^{(3)}(\mathbf{x})} \right]$. CT ventilation is premised on the ability to infer voxel volume changes induced by the spatial mapping $\phi$. Multivariate calculus (the “Change of Variables” Theorem) dictates that the determinant of the Jacobian, often referred to as the Jacobian factor, represents the magnification factor for volumes under the transformation $\phi$:

2 $$\begin{aligned}&\mathrm{Vol}(\hat{{\Omega }})=\int \limits _{\bar{{\Omega }}}{\left| {\det \left( {J(\mathbf{x};\;\mathbf{d})} \right) } \right| \;\hbox {d}{} \mathbf{x}}, \end{aligned}$$

3 $$\begin{aligned}&J(\mathbf{x};\;\mathbf{d})=\left[ {{\begin{array}{ccc} {1+\frac{\partial d^{(1)}(\mathbf{x})}{\partial x_1 }}&{} {\frac{\partial d^{(1)}(\mathbf{x})}{\partial x_2 }}&{} {\frac{\partial d^{(1)}(\mathbf{x})}{\partial x_3 }} \\ {\frac{\partial d^{(2)}(\mathbf{x})}{\partial x_1 }}&{} {1+\frac{\partial d^{(2)}(\mathbf{x})}{\partial x_2 }}&{} {\frac{\partial d^{(2)}(\mathbf{x})}{\partial x_3 }} \\ {\frac{\partial d^{(3)}(\mathbf{x})}{\partial x_1 }}&{} {\frac{\partial d^{(3)}(\mathbf{x})}{\partial x_2 }}&{} {1+\frac{\partial d^{(3)}(\mathbf{x})}{\partial x_3 }} \\ \end{array}}}\right] , \end{aligned}$$

where $\bar{{\Omega }}$ is the initial reference domain and $\hat{{\Omega }}$ is the image $\bar{{\Omega }}$ of under the transformation $\phi$. Equation (2) assumes $\phi$ is differentiable and one-to-one [16]. Modern DIR algorithms compute diffeomorphic spatial transformations that satisfy these assumptions and further require the determinant of the Jacobian to be strictly positive [32, 33].

For computing CT ventilation, $\bar{{\Omega }}$ is taken to be a cube of unit volume centered on the voxel location $\mathbf{x}_{k}$ and, rather than compute the integral in Eq. (2), the volume of the deformed voxel is approximated (assuming $\phi$ is diffeomorphic) as

4 $$\begin{aligned} \mathrm{vol}(\hat{{\Omega }})\approx \det \left( {J(\mathbf{x}_\mathbf{k} ;\;\mathbf{d})} \right) \cdot \underbrace{\mathrm{Vol}(\bar{{\Omega }})}_{=1}=\det \left( {J(\mathbf{x}_\mathbf{k};\;\mathbf{d})}\right) \end{aligned}$$

Equation (4) is exact when the Jacobian is constant, or equivalently, when the transformation $\phi$ is affine on $\bar{{\Omega }}$. An estimate for specific volume change, $V(\mathbf{x}_{k})$, can be defined from Eq. (4) as:

5 $$\begin{aligned} V(\mathbf{x}_k )=\det \left( {J(\mathbf{x}_{k}; \mathbf{d})}\right) -1\approx \mathrm{Vol}(\hat{{\Omega }})-\mathrm{Vol}(\bar{{\Omega }}). \end{aligned}$$

Equation (5) is a commonly employed ventilation metric with the demonstrated potential to quantify lung function [15, 23]. A transformation-based CT ventilation image is computed from a DIR displacement field by evaluating Eq. (5) for all lung voxels. In general, this process requires first computing numerical approximations to the first-order displacement field derivatives that define the Jacobian. For example, the forward difference approximation (under the unit voxel assumption) is defined as:

6 $$\begin{aligned} \frac{\partial d^{(i)}(\mathbf{x}_k )}{\partial x_j }\approx d^{(i)}(\mathbf{x}_k +\mathbf{e}_j )-d^{(i)}(\mathbf{x}_k ), \end{aligned}$$

where $\mathbf{e}_{j}$ is the standard basis vector. The corresponding forward difference ventilation metric is defined as:

7 $$\begin{aligned}&\tilde{V}(\mathbf{x}_k)=\det \left( {\tilde{J}(\mathbf{x}_k ;\;\mathbf{d}}\right) -1, \end{aligned}$$

8 $$\begin{aligned}&\tilde{J}(\mathbf{x}_k ;\;\mathbf{d})=\left[ {{\begin{array}{c@{\quad }c@{\quad }c} {1+d^{(1)}(\mathbf{x}_k +\mathbf{e}_1 )-d^{(1)}(\mathbf{x}_k )}&{} {d^{(1)}(\mathbf{x}_k +\mathbf{e}_2 )-d^{(1)}(\mathbf{x}_k )}&{} {d^{(1)}(\mathbf{x}_k +\mathbf{e}_3 )-d^{(1)}(\mathbf{x}_k )} \\ {d^{(2)}(\mathbf{x}_k +\mathbf{e}_1 )-d^{(2)}(\mathbf{x}_k )}&{} {1+d^{(2)}(\mathbf{x}_k +\mathbf{e}_2 )-d^{(2)}(\mathbf{x}_k )}&{} {d^{(2)}(\mathbf{x}_k +\mathbf{e}_3 )-d^{(2)}(\mathbf{x}_k )} \\ {d^{(3)}(\mathbf{x}_k +\mathbf{e}_1 )-d^{(3)}(\mathbf{x}_k )}&{} {d^{(3)}(\mathbf{x}_k +\mathbf{e}_2 )-d^{(3)}(\mathbf{x}_k )}&{} {1+d^{(3)}(\mathbf{x}_k +\mathbf{e}_3 )-d^{(3)}(\mathbf{x}_k )} \\ \end{array}}}\right] . \end{aligned}$$

## Perturbation analysis of the ventilation metric

Reproducibility is a key metric for assessing the clinical utility of an imaging modality. Given that transformation-based CT ventilation is derived from DIR, ideally, similar DIR transformations should generate similar ventilation images. Thus, investigating how small changes in the DIR displacement field affect the ventilation metric in Eq. (7) (or one derived by another finite differencing scheme) is key to understanding the numerical stability, and consequently, the reproducibility of CT ventilation. In general, a numerical analysis with respect to the entire displacement field is difficult due to the nonlinearity of the determinant calculation. However, with respect to a single voxel $\mathbf{x}_k$, the sensitivity of the ventilation metric can be described in terms of a perturbation, $\mathbf{h}$, to the displacement vector $\mathbf{d}(\mathbf{x}_k )$.

**Fig. 1:**
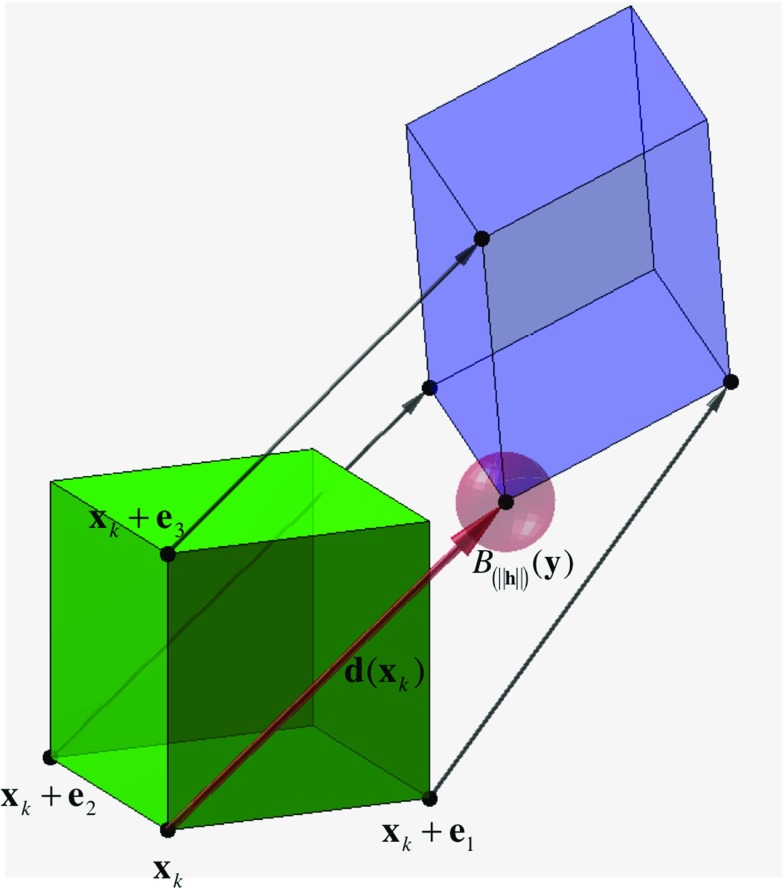
Undeformed voxel (*green*) is mapped by the displacement vectors to create the deformed voxel (*purple*). Equation (10) describes how the ventilation metric varies when $\mathbf{d}(\mathbf{x}_k)$ is perturbed by a vector with magnitude $\left\| \mathbf{h}\right\|$. The perturbation implies that the mapped position $\mathbf{y}=\mathbf{x}_k +\mathbf{d}(\mathbf{x}_k )-\mathbf{h}$ lies within the *red ball* of radius of $\left\| \mathbf{h} \right\|$ centered on the unperturbed position, denoted $B_{\left\| \mathbf{h} \right\| } (\mathbf{y})$

Figure 1 illustrates the undeformed voxel, the deformed voxel, and the corresponding displacement vectors needed to compute both the forward difference Jacobian approximation and the ventilation metric $\tilde{V}(\mathbf{x}_k )$. Applying a perturbation $\mathbf{h}\in {\mathbb {R}}^{3}$ to $\mathbf{d}(\mathbf{x}_k )$ implies that the mapped position, $\mathbf{y}=\mathbf{x}_k +\mathbf{d}(\mathbf{x}_k )-\mathbf{h}$, lies within a ball of radius $\left\| \mathbf{h} \right\|$ centered on the unperturbed position $\mathbf{x}_k +\mathbf{d}(\mathbf{x}_k )$. The value of the ventilation metric, $V_\mathbf{h}$, corresponding to the perturbed displacement is computed by replacing $\mathbf{d}(\mathbf{x}_k)$ with $\mathbf{d}(\mathbf{x}_k )-\mathbf{h}$ in Eq. (8):

9 $$\begin{aligned} V_\mathbf{h} =\det \left( {\tilde{J}(\mathbf{x}_k ,\;\mathbf{d})+\;\left[ {{\begin{array}{c@{\quad }c@{\quad }c} {h^{(1)}}&{} {h^{(1)}}&{} {h^{(1)}} \\ {h^{(2)}}&{} {h^{(2)}}&{} {h^{(2)}} \\ {h^{(3)}}&{} {h^{(3)}}&{} {h^{(3)}} \\ \end{array}}}\right] } \right) \;-1. \end{aligned}$$

The difference between the perturbed and unperturbed ventilation metric values is bounded (see “Appendix” for derivation):

10 $$\begin{aligned} \left| {V_\mathbf{h} -\tilde{V}(\mathbf{x}_k )} \right| \le \frac{3\sqrt{3}}{2}\left\| \mathbf{h} \right\| \mathop {\cdot \max }\nolimits _{i} {\mathop {\prod }\limits _{\begin{array}{c} j=1\\ j\ne i \end{array}}^{3}}{\left( {1+\left\| {\nabla d^{(j)}(\mathbf{x}_k )}\right\| }\right) }, \end{aligned}$$

where

11 $$\begin{aligned} \nabla \tilde{d}^{(j)}(\mathbf{x}_k )=\left[ {{\begin{array}{l} {d^{(j)}(\mathbf{x}_k +\mathbf{e}_1 )-d^{(j)}(\mathbf{x}_k )} \\ {d^{(j)}(\mathbf{x}_k +\mathbf{e}_2 )-d^{(j)}(\mathbf{x}_k )} \\ {d^{(j)}(\mathbf{x}_k +\mathbf{e}_3 )-d^{(j)}(\mathbf{x}_k )} \\ \end{array}}}\right] . \end{aligned}$$

In other words, the result in Eq. (10) describes the potential change in the Eq. (7) ventilation metric, evaluated at a voxel $\mathbf{x}_k$, caused by slightly changing the value of the displacement vector $\mathbf{d}(\mathbf{x}_k )$. Though the analysis is limited to a single voxel, Eq. (10) implies that the sensitivity of the ventilation metric with respect to a single perturbation depends on the smoothness of the displacement field, i.e., large magnitude displacement field gradients amplify the effect of the perturbation. For a transformation with displacement gradients close to zero, such as a rigid shift, the change in ventilation metric is bounded by the perturbation magnitude. However, respiratory-induced lung motion is known to be nonlinear and non-uniform. These properties can result in larger magnitude displacement field gradients, and consequently, a higher potential perturbation impact on the ventilation metric. A magnitude change on the order of $\left\| \mathbf{h} \right\|$ is therefore a conservative estimate for lung CT DIR, given that the gradient magnitudes of the displacement field are likely to be greater than one [34].

## Computing spatial transformations with user-defined Jacobian values

Equation (10) is a bound on the maximum change a single displacement vector perturbation can induce on the ventilation metric. It essentially describes the maximum ventilation change possible with respect to a single perturbation. However, Eq. (10) suggests that changing a displacement value by 1.0 (voxels) can result in a ventilation metric change of 1.0. Considering that the Jacobian is the magnification factor of a volume under a spatial transformation, the difference between a Jacobian factor of 1.0 and 2.0 is the difference between zero volume change and a 100% volume increase (a Jacobian factor of 2.0 doubles the reference volume). This characteristic suggests that transformation-based CT ventilation is numerically unstable in that small changes to the displacement field can potentially result in large relative changes in the ventilation image. In order to test this hypothesis, we employ a novel numerical optimization method for computing a DIR spatial transformation that generates a user-defined Jacobian image. The method is described in Sections 3.1 and 3.2. In “Computing spatial transformations with user-defined Jacobian values” section, we use the method to (1) manufacture several different DIR transformations for the same inhale/exhale CT image pair and (2) demonstrate that DIR transformations with similar spatial accuracies are not guaranteed to produce similar CT ventilation images.

### Optimization formulation

Since the Jacobian provides a concise mathematical description for volume change under a spatial transformation, it stands to reason that this quantity can be controlled within a DIR framework. For example, constraining the Jacobian to be strictly positive would guarantee a diffeomorphic DIR solution [32, 33, 35]. Similarly, upper bounds designed to force all lung voxel volumes to decrease could be used to model inhale-to-exhale lung motion as a strict contraction.

Considering the nature of anatomical motion, modern DIR methods are designed to produce diffeomorphic transformations that prohibit physically unrealistic tissue folding [32, 33]. Requiring the Jacobian factor to be strictly positive for every voxel within the image domain (or connected region on interest), $\Omega$, enforces this constraint. Thus, the concept of generating a transformation that yields volume changes defined by a given positive function, $f(\mathbf{x}):{\mathbb {R}}^{3}\rightarrow {\mathbb {R}}^{+},$ is simply a generalization of the diffeomorphic constraint:

12 $$\begin{aligned} \det \left( {J(\mathbf{x};\;\mathbf{d})} \right) =f(\mathbf{x}),\forall \mathbf{x}\in \Omega . \end{aligned}$$

Intensity-based deformable image registration represents an ill-posed, nonlinear, nonconvex numerical optimization problem [1]. Thus, explicitly incorporating the equality constraints represented by Eq. (12) into a DIR formulation, as done in [36] for inequality constraints, increases the high computational complexity associated with DIR of volumetric images [37]. Instead, we propose a post-DIR processing approach that assumes the existence of a priori fidelity data, provided by a separate DIR method, to remove the need for optimizing an image similarity metric.

Specifically, for each voxel location

13 $$\begin{aligned} \mathbf{x}_k \in \Omega ,\;\;k=1,2,\;\ldots \;N\hbox {,}\;\;N=\left| \Omega \right| , \end{aligned}$$

we introduce the discretized variables, $$d_k^j $$, representing the three components of the unknown displacement vectors $$\mathbf{d}(\mathbf{x}_k )$$:

14 $$\begin{aligned} \mathbf{d}(\mathbf{x}_k )=\left[ {{\begin{array}{lll} {d_k^{(1)} }&{} {d_k^{(2)} }&{} {d_k^{(3)} } \\ \end{array} }} \right] ^{T}. \end{aligned}$$

Given a set of fidelity data $\mathbf{y}_i$, representing a priori displacement estimates for voxel locations specified by the index set *I*:

15 $$\begin{aligned} \mathbf{y}_{i} \approx \mathbf{d}(\mathbf{x}_i ),\;\;\forall i\in I,\;\;\;\left| I \right| =M, \end{aligned}$$

the goal is to determine a smooth displacement field that satisfies the Jacobian equality constraints and ideally is in agreement with the fidelity data. However, there is one Jacobian constraint for each of the *N* voxels in $\Omega$, and no assumptions placed on the number of displacement estimates, *M*. Thus, the problem is *ill-posed* in that the total number of equations provided by the fidelity data and the constraints, $3M+N$, is not guaranteed to be greater than the total number of unknowns in the displacement field, 3*N* [38]. Thus, we introduce Laplacian regularization in order to impose a degree of smoothness (determined by a parameter $\alpha$) on the solution displacement field and formulate a well-posed optimization problem [39]:

16 $$\begin{aligned}&\mathop {\min }\limits _\mathbf{d}\sum _{i\in I}{\left\| {\mathbf{d}(\mathbf{x}_i )-\mathbf{y}_i}\right\| }^{2}+\alpha \sum _{j=1}^{3}{\left\| {A{{\tilde{\mathbf{d}}}}_j}\right\| },\nonumber \\&\quad \hbox {such that} \quad \det \left( {\tilde{J}(\mathbf{x}_k ;\;\mathbf{d})}\right) =f(\mathbf{x}_k ),\;\forall \mathbf{x}_k \in \Omega . \end{aligned}$$

The matrix *A* represents the Laplacian operator:

17 $$\begin{aligned} \Delta d^{(j)}(\mathbf{x})=\frac{\partial ^{2}d^{(j)}}{\partial x_1^2 }+\frac{\partial ^{2}d^{(j)}}{\partial x_2^2 }+\frac{\partial ^{2}d^{(j)}}{\partial x_3^2}, \end{aligned}$$

discretized by centered finite differences (7-point stencil) under a zero-normal derivative boundary condition. The vectors ${\tilde{\mathbf{d}}}_j =\left[ {d_1^{(j)} \;d_2^{(j)} \;\cdots \;d_N^{(j)}}\right] ^{T}$ organize the discretized displacement variables for each spatial dimension lexicographically and in correspondence with the Laplacian discretization.

The optimization problem defined by Eq. (16) is comprised of a linear least squares objective function with nonlinear equality constraints. Without the Jacobian constraints, Eq. (16) is simply an overdetermined linear least squares formulation for computing a smooth displacement field from fidelity data:

18 $$\begin{aligned} \mathop {\min }\limits _\mathbf{d} \sum _{i\in I} {\left\| {\mathbf{d}(\mathbf{x}_i )-\mathbf{y}_i}\right\| ^{2}+\alpha \sum _{j=1}^3 {\left\| {A{\tilde{\mathbf{d}}}_j}\right\| ^{2}.}} \end{aligned}$$

While Eq. (18) has a unique solution, proving the existence or uniqueness of a solution for an equality constrained nonlinear, nonconvex optimization problem, such as Eq. (16), is not trivial [40]. For the purposes of this study, we operate under the assumption that a solution exists and mention that these issues will be further explored in future work. A numerical solution to problem (16) is computed using the well-known augmented Lagrangian method (see [40, 41] for full derivation and convergence analysis).

**Table 1:** Properties of the inhale/exhale CT image pair (Case 6 from www.dir-lab.com) used for the constrained Jacobian experiments are given in the first column

Image properties	Spatial transformation	Average mm error (Std.)	Max mm error	Average Jacobian value (std.)
Size (voxels): 512 $\times$ 512 $\times$ 128	No DIR	11.10 (6.98)	27.59	
Voxel dimensions (MM): 0.97 $\times$ 0.97 $\times$ 2.5	Unconstrained DIR	0.99 (0.99)	5.37	0.79 (0.11)
Number of landmarks: 419	Contraction constraint: LB $=$ 0.5, UB $=$ 1.00	1.08 (1.02)	5.00	0.79 (0.08)
	Contraction constraint: LB $=$ 0.5, UB $=$ 0.75	1.66 (1.16)	5.71	0.73 (0.03)
	Forced spatial correlation with SPECT Perfusion	1.47 (1.16)	5.46	0.79 (0.03)

The spatial accuracy results for the unconstrained DIR solution and each of three constrained DIR examples are given in the remaining columns. For all experiments, the average millimeter (MM) error stays below the axial slice spacing

### Constructing Jacobian constraint functions

The solution to Eq. (16) is a DIR spatial transformation whose Jacobian values are equal to those specified by *f*. In order to apply this method, one must first explicitly define $f(\mathbf{x})$: the Jacobian factor value for each voxel within the region of interest. However, the existence of a priori fidelity data implies that the unique unconstrained solution, $\mathbf{d}^{\mathrm{unc}}$, can be computed by solving the least squares problem in Eq. (18). Under this framework, a function *f* possessing desired physical properties, such as smoothness, volume preservation, or spatial structure can be defined as a function of the unconstrained Jacobian values, $J(\mathbf{x};\;\mathbf{d}^{\mathrm{unc}})$. For instance, a smoother variant of the unconstrained Jacobian image can be obtained by applying a Gaussian convolution filter with variance $\sigma$:

19 $$\begin{aligned} f(\mathbf{x})=G_\sigma {*}\det \left( {J(\mathbf{x};\;\mathbf{d}^{\mathrm{unc}})} \right) . \end{aligned}$$

More general Jacobian constraints of the form:

20 $$\begin{aligned} \mathrm{LB}\le \det \left( {J(\mathbf{x};\;\mathbf{d})}\right) \le \mathrm{UB},\quad \forall \mathbf{x}\in \Omega , \end{aligned}$$

result in a diffeomorphic *contraction* transformation (every voxel either shrinks, or maintains volume under the transformation) when $\mathrm{LB}\ge 0$ and $\mathrm{UB}=1$. Similarly, $\mathrm{UB}>\mathrm{LB}\ge 1$ results in an *expansion*. A general bound constraint function can be defined as:

21 $$\begin{aligned} f(\mathbf{x})=G_\sigma {*}\hat{{f}}(\mathbf{x};\;\mathbf{d}^{\mathrm{unc}},\;\mathrm{LB},\;\mathrm{UB}), \end{aligned}$$

where

22 $$\begin{aligned}&\hat{{f}}(\mathbf{x};\;\mathbf{d}^{(unc)},\mathrm{LB},\mathrm{UB})\nonumber \\&\quad =\left\{ {{\begin{array}{c@{\quad }c@{\quad }c} {\det \left( {J(\mathbf{x};\;\mathbf{d}^{\mathrm{unc}})}\right) ,}&{}\; {\hbox {if}}&{}\; {\mathrm{LB}\le \det \left( {J(\mathbf{x};\;\mathbf{d}^{\mathrm{unc}})}\right) \le \mathrm{UB}} \\ {\mathrm{LB},}&{}\;{\hbox {if}}&{}\; {\det \left( {J(\mathbf{x};\;\mathbf{d}^{\mathrm{unc}})}\right) <\mathrm{LB}}\\ {\mathrm{UB},}&{}\;{\hbox {if}}&{}\; {\det \left( {J(\mathbf{x};\;\mathbf{d}^{\mathrm{unc}})}\right) >\mathrm{UB}}\\ \end{array}}}\right\} .\nonumber \\ \end{aligned}$$

As mentioned in the Introduction, a common approach for validating CT ventilation is to determine the amount of spatial correlation between the ventilation image and an established functional imaging modality (i.e., SPECT ventilation, Hyperpolarized $\hbox {He}^{3}$ MRI). A constraint function enforcing a linear spatial correlation between the Jacobian/ventilation image and a functional image $g(\mathbf{x})$ requires defining the coefficients of a linear intensity mapping. This can be accomplished by first calculating the line of best (least squares) fit with respect to the unconstrained Jacobian values:

23 $$\begin{aligned} \mathop {\min }\limits _{a,b} \sum _{\mathbf{x}_k \in \Omega }{\left[ {ag(\mathbf{x})+b-\det \left( {J(\mathbf{x};\;\mathbf{d}^{\mathrm{unc}})}\right) } \right] ^{2},} \end{aligned}$$

where the optimal fit coefficients $a^{*},b^{*}$ are the solution to problem (23). The constraint function closest (in terms of least squares) to the unconstrained Jacobian that enforces a strict linear spatial correlation with $g(\mathbf{x})$ is defined as:

24 $$\begin{aligned} f(\mathbf{x})=a^{*}g(\mathbf{x})+b^{*}. \end{aligned}$$

In “Numerical experiments” section, the constraint functions defined by Eqs. (19), (21) and (24) are used to compute several DIR transformations for the same inhale/exhale CT image pair. While the spatial accuracies of the transformations are similar, we show that the corresponding ventilation images exhibit significantly different physical characteristics.

**Fig. 2:**
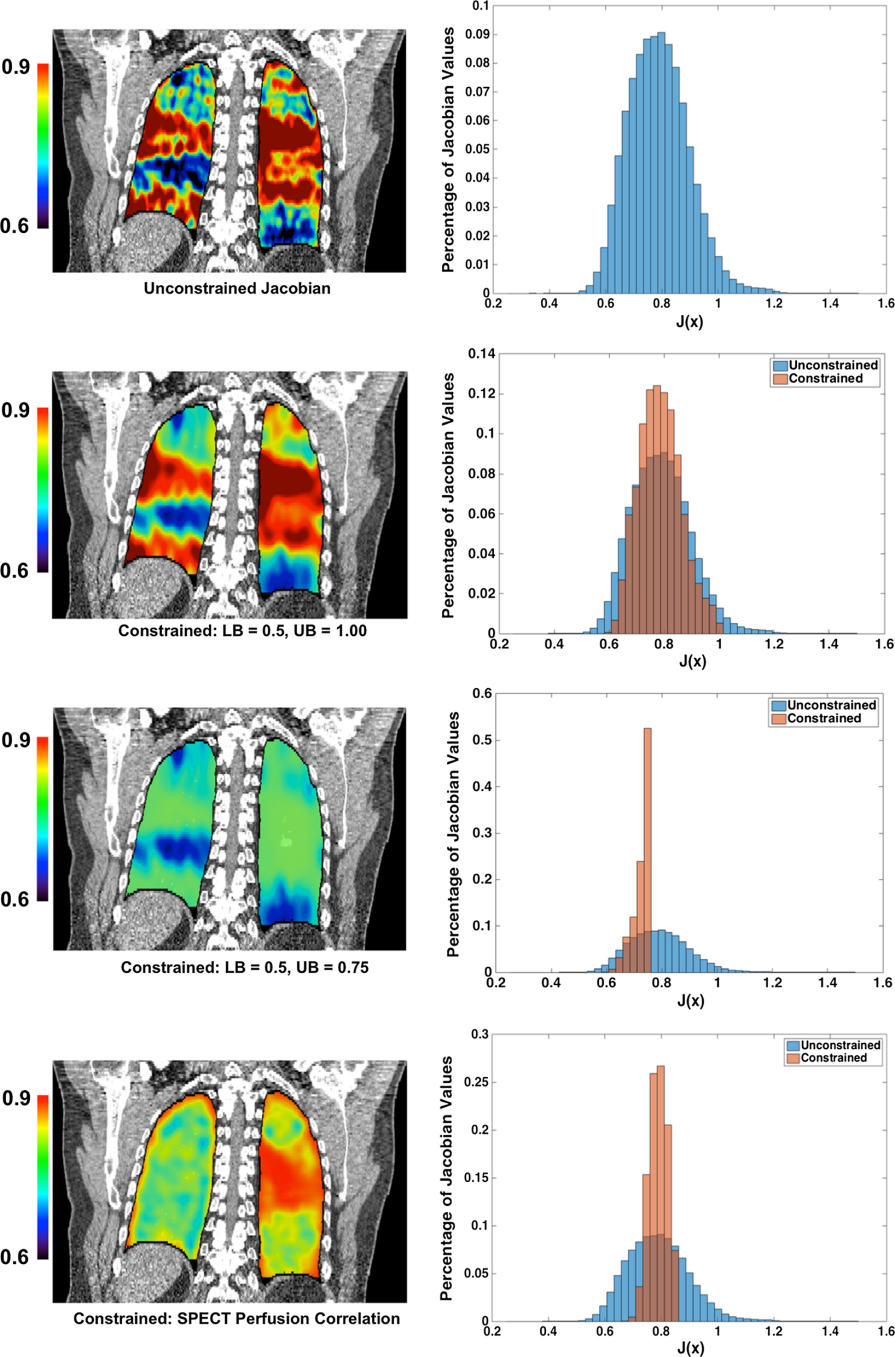
Coronal slice 293 from the four CT ventilation Jacobian images corresponding to the experiments described in Table 1 are shown in the *left column*. The intensity values indicate the Jacobian measured volume change for each voxel. In the *right column*, a histogram plot of all Jacobian values for each image ventilation image is shown, with the unconstrained histogram superimposed for reference. The results indicate that for the same inhale/exhale CT image pair, it is possible to compute transformation-based CT images with significantly different physical characteristics, despite the fact that the corresponding DIR solutions maintain subvoxel average mm accuracy

## Numerical experiments

The constrained Jacobian method (Eq. (16)) was used to assess the numerical stability of transformation-based CT ventilation imaging with respect to DIR spatial accuracy. We examine two constraint sets: (1) strict contraction constraints and (2) enforced linear spatial correlation with a SPECT perfusion image. The inhale/exhale thoracic CT image pair listed as “Case 6” on the www.dir-lab.com image repository was used for all experiments. Spatial accuracy, with respect to expert-determined landmark point pairs (also available on the dir-lab website), was quantified as the Euclidian distance in millimeters between the landmark positions and the positions mapped by the DIR. The image and landmark characteristics are summarized in Table 1 and detailed in [42].

An unconstrained DIR solution for the test case was computed using the MILO algorithm [26]. The algorithm achieved an average millimeter (standard deviation) error of 0.99 (0.99). The unconstrained Jacobian image was then used to compute two bound constraint functions according to Eq. (22) with parameters detailed in Table 1. The constrained Jacobian optimization method Eq. (16) was then used to compute (with the unconstrained MILO DIR solution serving as the fidelity data) spatial transformations satisfying the two sets of contraction bounds (shown in Fig. 2). The spatial accuracies of the resulting DIR transformations are listed in Table 1, and the histogram of Jacobian values for each experiment is given in Fig. 2. The results demonstrate that while the average Jacobian value did not vary greatly across the different DIR transformations ([0.73 0.79]), the distribution of the Jacobian values varied with standard deviations between 0.03 and 0.11. This variation resulted in different ventilation estimates. In particular, the two upper bound constraints, UB $=$ 0.75 and UB $=$ 1.00, represent a 25% difference in the minimum volume change and ventilation metric for each voxel. The histogram plots given in Fig. 2 illustrate this difference. However, the average mm errors of the two DIR solutions (1.08 and 1.66 respectively) both remain well below the axial slice spacing of 2.5mm.

**Fig. 3:**
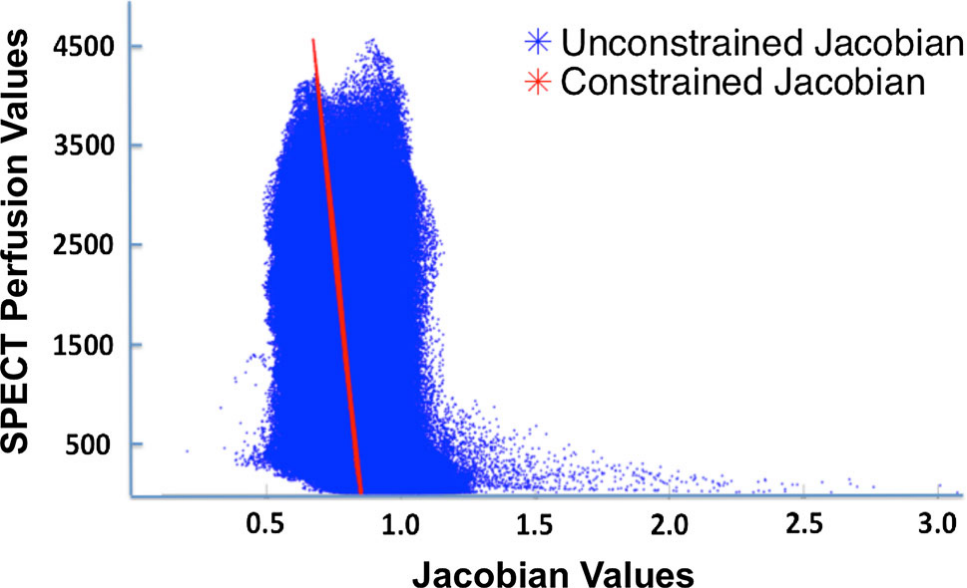
A plot of the unconstrained DIR Jacobian values (*x-axis*) versus the spatially corresponding SPECT Perfusion values (*y-axis*) is given in *blue*. The correlation between the two datasets is $-0.29$. The constrained Jacobian values are also plotted (*red*). The spatial correlation between the constrained values and the SPECT values is $-1.00$. Despite these seemingly significant differences, the average mm spatial error of the corresponding DIR solutions is 0.99 and 1.47 mm, respectively, both of which are well below the axial slice spacing of the CT image pairs (2.5 mm)

In a previous study, we demonstrated the correlation between 4DCT-derived ventilation defects and defects within SPECT pulmonary perfusion images caused by malignant airway stenosis [17]. The www.dir-lab.com test Case 6 used in this study was included in the imaging dataset used for study [17]. After performing an affine registration to align the SPECT perfusion image with the unconstrained CT ventilation image (defined on the T00 maximum inhale phase), we then calculated the spatial correlation between the unconstrained Jacobian values and the SPECT perfusion values to be $-0.29$. A plot of spatially corresponding unconstrained Jacobian values versus SPECT perfusion values is given in Fig. 3. The linear transformation defined by Eq. (24) was then applied to the SPECT perfusion values to generate a Jacobian constraint function. The constrained Jacobian optimization method (with the unconstrained MILO DIR solution serving as the fidelity data) was used to compute a DIR transformation satisfying the constraint. The correlation between spatially corresponding constrained Jacobian values and SPECT perfusion values (plotted in Fig. 3) is $-1.00$, while the average mm error of the corresponding DIR transformation was 1.47 (1.16). Thus, it is possible to manufacture a DIR solution with subvoxel average mm error that perfectly correlates with a SPECT perfusion image.

## Discussion

CT-derived ventilation imaging is an emerging medical image analysis tool with demonstrated utility in disease diagnosis [43] and in quantifying radiotherapy dose response [11, 12]. Previous studies seeking to validate CT-derived ventilation imaging examine the correlation between ventilation values and the intensities of a functional imaging modality, such as SPECT ventilation [10], SPECT perfusion [17, 44], hyperpolarized $\hbox {He}^{3}$ MRI [19], or PET [18]. However, CT-derived ventilation is dependent on DIR computation. Consequently, sensitivity to DIR algorithm [22, 45], as well as to image characteristics that can influence DIR, such as 4DCT phase binning [23] and patient breathing variations [46, 47], are known issues that affect CT ventilation reproducibility [46]. The results and analysis in this paper only consider the uncertainty introduced by DIR, whereas the uncertainties in 4DCT image acquisition are certainly important but left outside the scope of this work.

Our novel methodology for computing spatial transformations with constrained Jacobian values provides a tool for assessing the sensitivity of transformation-based ventilation imaging with respect to DIR spatial accuracy. In accordance with the theoretical perturbation bound given by Eq. (10), the numerical results indicate that the constrained Jacobian method can produce manufactured ventilation images with significantly different physical properties while maintaining a DIR spatial accuracy below the CT axial slice spacing.

The strict contraction constraints provide an example where DIR spatial accuracy is not overly sensitive to large changes in the Jacobian. Specifically, a strict contraction defined by Eq. (22) with LB $=$ 0.5 and UB $=$ 1.00 was computed from the unconstrained DIR solution at the cost of only a 0.10 change in the average mm error. In fact, the maximum magnitude error (over all landmarks) decreased using the strict contraction constraint. A more drastic constraint with LB $=$ 0.5 and UB $=$ 0.75 resulted in a larger decrease in spatial accuracy. This phenomenon is intuitive and in agreement with the analysis of the perturbation bound (10): The magnitude difference in spatial accuracy between two displacement fields is on the order of the magnitude difference between the corresponding Jacobian images. However, as illustrated by the Jacobian value histograms in Fig. 2, a displacement perturbation magnitude of 1.0 voxels only marginally affects spatial accuracy, whereas a Jacobian value change of +1.0 represents at minimum a 100% change for contracting voxels ($J(\mathbf{x})\le 1.00)$. As a consequence of this relationship, the distribution of Jacobian values can vary widely between DIR transformations with spatial accuracies that are on the order of the voxel spacing. Moreover, in practice, DIR spatial accuracies between competing methods could potentially be worse, which would in turn further increase the magnitude of the potential variation. Thus, as previous studies have reported, transformation-based ventilation imaging is difficult to reproduce [47] and voxel volume change measurements can significantly vary between different DIR algorithms [21]. In all three experiments (Table 1), the resulting spatial accuracy of the constrained DIR maintained an average mm error well below the axial slice spacing of 2.5 mm. Moreover, the maximum mm error remained close to constant across all experiments. These results imply that transformation-based ventilation imaging is not stable with respect to the DIR, i.e., small changes to the DIR transformation can potentially result in large changes in the Jacobian image. For all experiments, the average mm error ranged from 0.99 (0.99) to 1.66 (1.16) but yielded drastically different Jacobian images, as shown by the histograms in Fig. 2.

The constrained Jacobian methodology also provides an alternative approach to traditional multi-modality comparison-based ventilation validation studies. Since correlation between the ventilation image and a target functional image can be manufactured, as demonstrated by the SPECT perfusion example shown in Figs. 2, 3, determining the degree to which DIR-based ventilation is related to the functional image reduces to examining the spatial accuracy of the resulting constrained DIR transformation. For example, if the magnitude DIR change required to force a desired correlation between the unconstrained ventilation image and the functional image is greater than a predetermined threshold, the likelihood is that CT ventilation does not relate well with the given functional image. Similarly, a small magnitude change would imply that correlation is likely. Ultimately, a concise probabilistic definition of the “true” DIR solution is needed to accurately describe the uncertainty in the CT ventilation calculation. For instance, one could define a statistical model describing the likelihood of all ventilation images generated from spatial transformations within a given neighborhood of a DIR solution (with respect to a specified norm and function space). A similar idea based on a Bayesian statistical framework has been proposed for assessing DIR spatial accuracy with landmark point pairs [48].

The ability to manufacture user-defined (transformation-based) CT ventilation images has not been addressed within previous application or validation studies and should be taken into consideration when interpreting past results. However, ventilation imaging computed from HU is difficult to constrain since the measured volume changes depend on variations in HU values between the corresponding inhale/exhale voxels determined by the DIR. Describing the mismatch between the target volume change and HU volume change is essentially an image similarity metric. The resulting mathematical formulation for computing an HU-constrained transformation would look similar to an intensity-based DIR formulation and would require overcoming the same computational difficulties, such as nonlinearities, discontinuities, and large problem size, in order to compute a solution. Ideally, the transformation-based and HU-based ventilation images should be equivalent since they represent two ways of measuring the same quantity. Though certainly possible, a DIR formulation enforcing this constraint would represent a formidable computational challenge.

The goal in developing CT ventilation is to provide an alternative to existing nuclear medicine- or MR-based functional imaging modalities. As such, previous clinical validation studies have focused on assessing the correlation between CT ventilation and various other established modalities. While this validation strategy is useful for assessing physiological fidelity on a qualitative level, these modalities typically quantify the distribution of inhaled gas tracers and therefore provide no direct measurements of regional volume changes. As such, a mechanism for precisely quantifying the physiological accuracy of CT ventilation is currently lacking. In the absence of a ground truth dataset, it is reasonable to instead assess the quality of the employed DIR solution, a process for which ground truth validation (based on expert-determined landmark point pairs) does exist. Ideally, the most spatially accurate DIR solution should generate the most physiologically accurate CT ventilation image. However, as the analysis and results from “Perturbation analysis of the ventilation metric and Numerical experiments” sections indicate, there can be large variability between the ventilation images generated by similarly accurate DIR solutions. This implies that high DIR spatial accuracy is a necessary condition for accurate CT ventilation, but it is not sufficient. Thus, incorporating additional information or constraints into the CT ventilation model could reduce the amount of variability. For instance, one could require the DIR solution to respect a pulmonary biomechanical model. In essence, this type of approach reduces the size of the DIR solution set, which intuitively would have the effect of reducing CT ventilation variability. Moreover, a second layer of validation could be designed if, in addition to regional volume change estimates, the biomechanical model produced clinically quantifiable outputs such as estimated spirometry or pulmonary function test data. Quantitative clinical validation of CT ventilation will be an area of our future research.

## Conclusion

This work investigates the numerical stability of transformation-based CT ventilation. We mathematically prove that the maximum change in ventilation metric that can be induced by a perturbation to a single displacement vector is on the order of perturbation magnitude. Considering the disproportionate scaling between DIR displacement magnitudes and Jacobian magnitudes, this result suggests that transformation-based CT ventilation is numerically unstable, i.e., small changes to the displacement field can potentially result in large relative changes in the ventilation image. In order to test this hypothesis, we also presented a numerical method for computing a DIR transformation that generates a user-defined CT ventilation image. This method was employed to create four different DIR solutions for the same inhale/exhale CT image pair. Even though all four DIR solutions demonstrated subvoxel average spatial accuracy, the functional information depicted by the four corresponding CT ventilation images varied substantially. In particular, we generated a spatially accurate DIR solution such that its Jacobian values correlate perfectly with a SPECT perfusion nuclear image. Future work in this area includes (1) leveraging the constrained Jacobian methodology to develop a Bayesian framework for quantifying the uncertainty in CT-derived ventilation imaging, (2) incorporating intensity-based volume changes into the constrained Jacobian formulation, and (3) developing approaches for the quantitative clinical validation of CT ventilation.

## Appendix

For a single voxel $\mathbf{x}_{k}$, the magnitude change in ventilation metric (Eq. (7)) induced by a perturbation, $\mathbf{h}\in {\mathbb {R}}^{3},$ to the displacement vector $\mathbf{d}(\mathbf{x}_k)$ is bounded according to Eq. (10). The bound is derived by first rewriting the perturbed ventilation metric in Eq. (9) as:

25 $$\begin{aligned} V_\mathbf{h} =\det \left( {\tilde{J}(\mathbf{x}_k ;\;\mathbf{d})+\mathbf{hz}^{T}} \right) -1, \end{aligned}$$

where $\mathbf{hz}^{T}$ is the outer product of the perturbation vector and $\mathbf{z}=[1,1,1]^{T}$. Applying the Matrix Determinant Lemma to Eq. (25) gives:

26 $$\begin{aligned} V_\mathbf{h}= & {} \det \left( {\tilde{J}(\mathbf{x}_k ;\;\mathbf{d})} \right) \left( {1+\mathbf{z}^{T}\tilde{J}(\mathbf{x}_k ;\;\mathbf{d})^{-1}\mathbf{h}} \right) -1,\nonumber \\= & {} \det \left( {\tilde{J}(\mathbf{x}_k ;\;\mathbf{d})} \right) +\mathbf{z}^{T}A\mathbf{h}-1, \end{aligned}$$

where

27 $$\begin{aligned} A=\det \left( {\tilde{J}(\mathbf{x}_k ;\;\mathbf{d})} \right) \tilde{J}(\mathbf{x}_k ;\;\mathbf{d})^{-1}, \end{aligned}$$

is the *Adjugate* of the matrix $\tilde{J}(\mathbf{x}_k ;\;\mathbf{d})$. Thus,

28 $$\begin{aligned} V_\mathbf{h} -\tilde{V}(\mathbf{x}_k)= & {} \underbrace{\det \left( {\tilde{J}(\mathbf{x}_k ;\;\mathbf{d})}\right) +\mathbf{z}^{T}A\mathbf{h}-1}_{V_\mathbf{h}}\nonumber \\&-\underbrace{\left[ {\det \left( {\tilde{J}(\mathbf{x}_k ;\;\mathbf{d})}\right) -1}\right] }_{\tilde{V}(\mathbf{x}_k )}=\mathbf{z}^{T}A\mathbf{h}, \end{aligned}$$

and

29 $$\begin{aligned} \left| {V_\mathbf{h} -\tilde{V}(\mathbf{x}_k )} \right| =\left| {\mathbf{z}^{T}A\mathbf{h}} \right| \le \left\| \mathbf{z} \right\| \;\;\left\| A \right\| \;\;\left\| \mathbf{h} \right\| \;, \end{aligned}$$

where $\Vert \!|\cdot \Vert$ is the $l_{2}$-norm. The 2nd *compound matrix* of $\tilde{J}^{T}(\mathbf{x}_k ;\;\mathbf{d})$, denoted $C_2 \left( {\tilde{J}^{T}(\mathbf{x}_k ;\;\mathbf{d})} \right)$, is the matrix whose elements are the determinants of all $2\times 2$ minors of $\tilde{J}^{T}(\mathbf{x}_k ;\;\mathbf{d})$. Furthermore, the adjugate matrix can be expressed as:

30 $$\begin{aligned} A=DC_2 \left( {\tilde{J}^{T}(\mathbf{x}_k ;\;\mathbf{d})} \right) D^{T},\;\hbox { with }D=\left[ {{\begin{array}{c@{\quad }c@{\quad }c} 1&{} 0&{} 0 \\ 0&{} {-1}&{} 0 \\ 0&{} 0&{} 1 \\ \end{array}}}\right] , \end{aligned}$$

and therefore bounded

31 $$\begin{aligned}&A\le \left\| D\right\| \;\;\left\| {C_2\left( {\tilde{J}^{T}(\mathbf{x}_k ;\;\mathbf{d})}\right) }\right\| \;\;\left\| {D^{T}} \right\| \nonumber \\&\quad =\left\| {C_2 \left( {\tilde{J}^{T}(\mathbf{x}_k ;\;\mathbf{d})}\right) }\right\| . \end{aligned}$$

Thus, according to Theorem 2.8 in [49]:

32 $$\begin{aligned}&\left\| A \right\| \le \left\| {C_2\left( {\tilde{J}^{T}(\mathbf{x}_k;\;\mathbf{d})}\right) } \right\| \nonumber \\&\quad <\left( {\frac{3}{2}} \right) \mathop {\max }\limits _{i} {\mathop {\prod }\limits _{\begin{array}{c} j=1\\ j\ne i \end{array}}^3} {\left\| {\hbox {Row}_j \left( {\tilde{J}(\mathbf{x}_k ,\mathbf{d})} \right) } \right\| } , \end{aligned}$$

with $\hbox {Row}_{i}\left( {\tilde{J}(\mathbf{x}_k ;\;\mathbf{d})}\right)$ equal to the $\hbox {i}{\mathrm{th}}$ row of the Jacobian matrix. Combining Eq. (32) with (29) yields:

33 $$\begin{aligned} \left| {V_\mathbf{h}-\tilde{V}(\mathbf{x}_k )} \right| \le \frac{3\sqrt{3}}{2}\left\| \mathbf{h} \right\| \mathop {\cdot \max }\limits _{i} {\mathop {\prod }\limits _{\begin{array}{c} j=1\\ j\ne i \end{array}}^{3}} {\left\| {\mathbf{e}_j +\nabla d^{(j)}(\mathbf{x}_k )} \right\| } ,\nonumber \\ \end{aligned}$$

where $\mathbf{e}_j$ is the standard basis vector. Finally, applying the triangle inequality to the norm in the product gives:

34 $$\begin{aligned} \left| {V_\mathbf{h} -\tilde{V}(\mathbf{x}_k )} \right| \le \frac{3\sqrt{3}}{2}\left\| \mathbf{h} \right\| \mathop {\cdot \max }\limits _{i} {\mathop {\prod }\limits _{\begin{array}{c} j=1\\ j\ne i \end{array}} ^{3}}{\left( {1+\left\| {\nabla d^{(j)}(\mathbf{x}_k )} \right\| }\right) }.\nonumber \\ \end{aligned}$$

Though this result is specific to a forward difference scheme, similar results can also be obtained for backward and centered difference schemes.
